# Supplementary material for: An E2-ubiquitin thioester-driven approach to identify substrates modified with ubiquitin and ubiquitin-like molecules
Source: Nat Commun. 2018 Nov 14;9:4776. doi: 10.1038/s41467-018-07251-5 (PMC6235928; doi:10.1038/s41467-018-07251-5)
Supplement: Supplementary file 1 — Supplementary Information [file 41467_2018_7251_MOESM1_ESM.pdf]

An E2-ubiquitin thioester-driven approach to identify substrates modified with ubiquitin and ubiquitin-like molecules

Bakos et al.,

Supplementary Information

Supplementary Figures

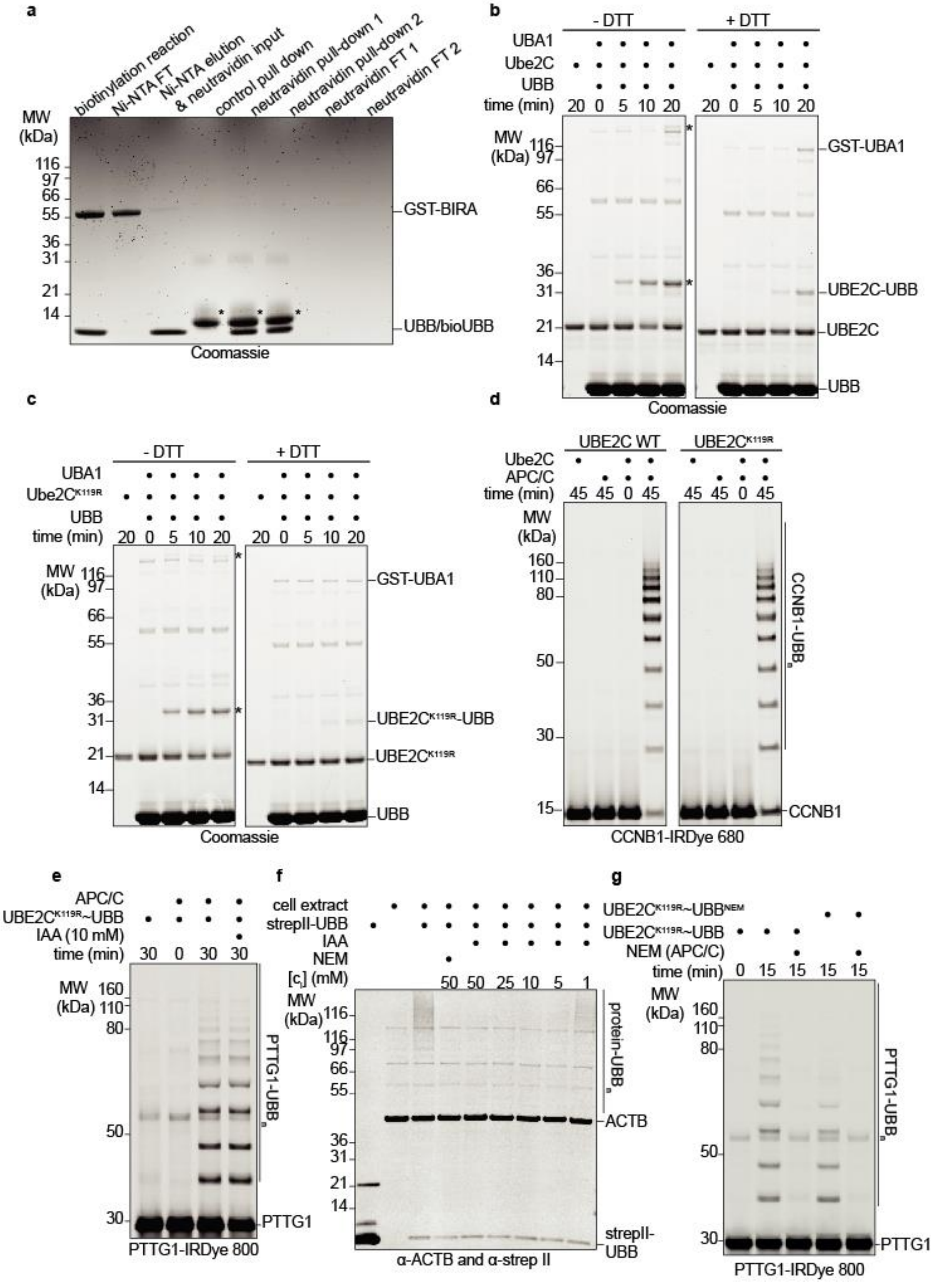

Supplementary Figure 1 | *In vitro* reconstitution of E2~dID. *(Figure legend on next page)*

(**a**) Representative SDS-PAGE (n=3) showing *in vitro* labeling and purification of biotinylated ubiquitin (bioUBB). Recombinant GST-BIRA and His-tagged avi-UBB were incubated for 4 hours at 30 ^{0}C followed by Ni-NTA purification of His-bioUBB to remove GST-BIRA. The degree of biotin labeling was assessed by monitoring the flow though (FT) of two independent NeutrAvidin pull-downs showing that all detectable UBB was immobilized on the beads. Asterisks indicate the NeutrAvidin monomer that is eluted by SDS-sample buffer and boiling. (**b**) Representative *in vitro* charging reaction (n=5) containing wild type UBE2C. The reactions were incubated for the indicated time, stopped by SDS sample buffer with or without DTT to monitor the degree of UBE2C~UBB thioester formation and were analyzed by SDS-PAGE. UBE2C-UBB species that are not sensitive to DTT treatment represent auto-ubiquitinated UBE2C. Asterisks indicate E1 and E2~UBB conjuagtes. (**c**) Analysis of representative *in vitro* charging reactions (n=5) as in (a) but a UBE2C^{K119R} mutant that exhibits a reduced auto-ubiquitination. Asterisks indicate E1 and E2~UBB thioesters. (**d**) Representative SDS-PAGE (n=2) and fluorescent scanning showing an *in vitro* APC/C activity assay based on purified components comparing the ability of wild type UBE2C and UBE2C^{K119R} to ubiquitinate an IRDye-680-labeled N-terminal fragment of CCNB1. (**e**) Representative SDS-PAGE (n=2) and fluorescent scan of *in vitro* APC/C activity assays comparing the ability of UBE2C^{K119R}~UBB from untreated and iodoacetamid (IAA)-treated charging reactions to drive ubiquitination of IRDye-800-labeled PTTG1. Note, the presence of IAA in the reaction does not interfere with APC/C activity. (**f**) Representative Western blot analysis (n=2) of an IAA titration assay in HeLa anaphase cell extracts. Note, all the extracts were treated with 10 µM MG132 to prevent ubiquitin-mediated protein proteolysis. (**g**) Representative SDS-PAGE (n=2) and fluorescent scan of *in vitro* APC/C activity assays comparing the ability of UBE2C^{K119R}~UBB from untreated or N-ethylmaleimide (NEM)-treated charging reactions to support ubiquitination of IRDye-800 labeled PTTG1 in the presence or absence of NEM-treated APC/C. Note, the presence of NEM does not interfere with the UBE2C^{K119R}~UBB activity.

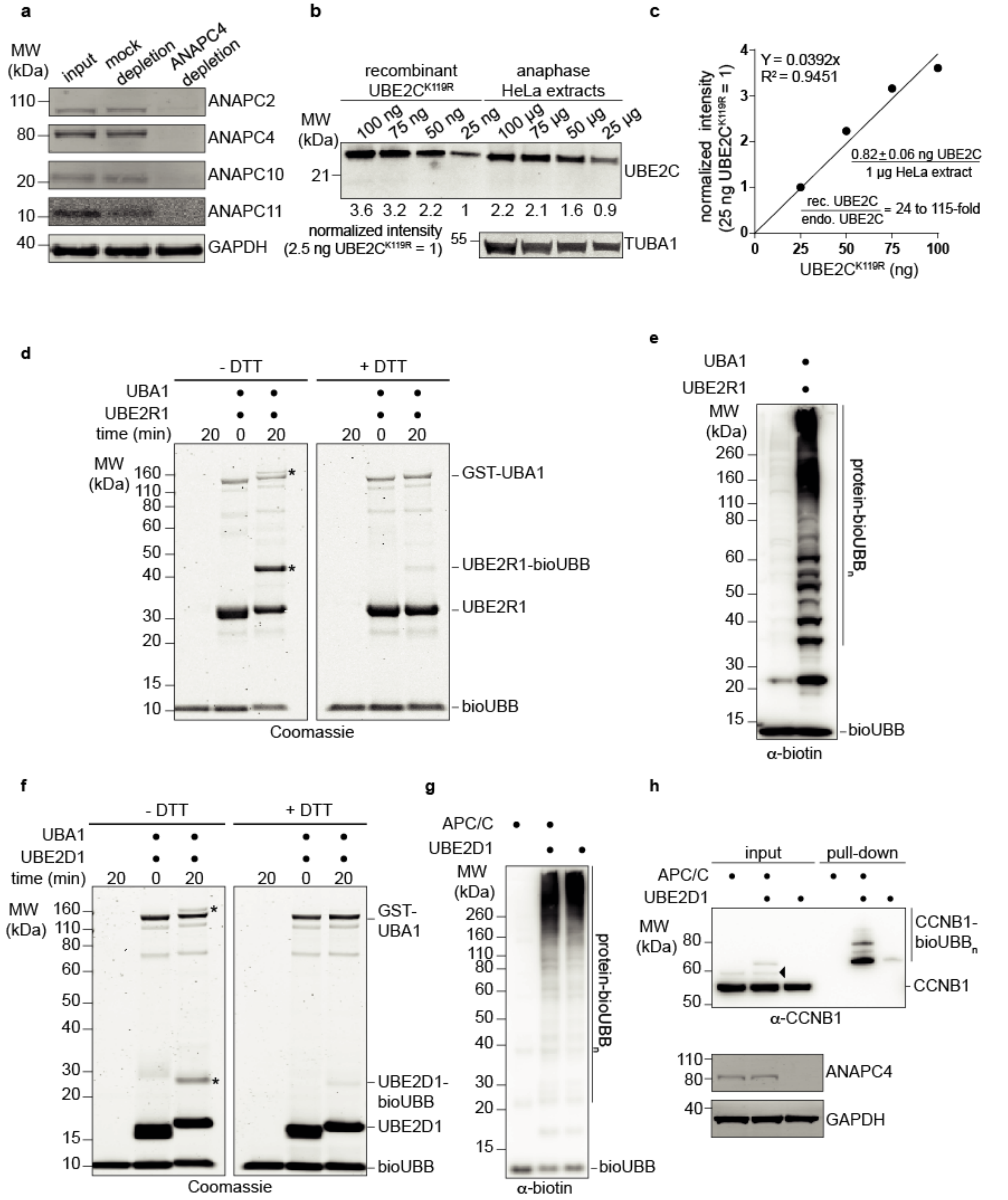

Supplementary Figure 2 | E2 and E3 protein levels during E2~dID and E2~dID with UBE2R1.

(Figure legend on next page)

(**a**) Representative Western blot (n=3) analysis of mock or ANAPC4-depleted HeLa anaphase cell extracts. Note, the ANAPC4 depletion also results in co-depletion of APC/C subunits forming the enzyme's active core. (**b** and **c**) Representative Western blot analysis (n=2) showing a titration of recombinant UBE2C^{K119R} and endogenous UBE2C present in the extract. The resulting UBE2C^{K119R} titration curve was used to estimate endogenous UBE2C levels in HeLa anaphase extracts and the degree of excess UBE2C^{K119R} added during E2~dID. (**d**) Representative *in vitro* charging reaction (n=5) containing UBE2R1. The reactions were incubated for the indicated time, stopped by SDS sample buffer with or without DTT to monitor the degree of UBE2R1~bioUBB thioester formation and were analyzed by SDS-PAGE. UBE2R1-bioUBB species that are not sensitive to DTT treatment represent auto-ubiquitinated UBE2R1. Asterisks indicate E1 and E2~bioUBB conjugates. (**e**) Representative Western blot analysis (n=2) showing UBE2R1~bioUBB-dependent covalent linkage of bioUBB molecules to proteins present in extracts of IAA-inactivated asynchronously growing retina pigment epithelial cells (hTERT RPE-1). Note, ubiquitination of proteins in the extract strictly depends on UBE2R1~bioUBB thioesters. (**f**) Representative *in vitro* charging reaction (n=4) as in (d) containing UBE2D1. UBE2D1-bioUBB species that are not sensitive to DTT treatment represent auto-ubiquitinated UBE2D1. Asterisks indicate E1 and E2~bioUBB conjugates. (**g**) Representative Western blot analysis (n=2) of E2~dID-dependent labeling of APC/C substrates with bioUBB in extracts. Note, while ubiquitination depends on UBE2D1~bioUBB thioesters it is not strongly impacted by the ANAPC4 depletion (compare + and - APC/C). (**h**) Representative E2~dID analysis (n=2) with UBE2D1 showing APC/C-dependent ubiquitination of CCNB1. After E2~dID bioUBB-modifed proteins were purified by NeutrAvidin beads and analyzed by Western blot. Note, CCNB1 species modified with endogenous ubiquitin are present in mitotic extracts when APC/C is present (arrow head).

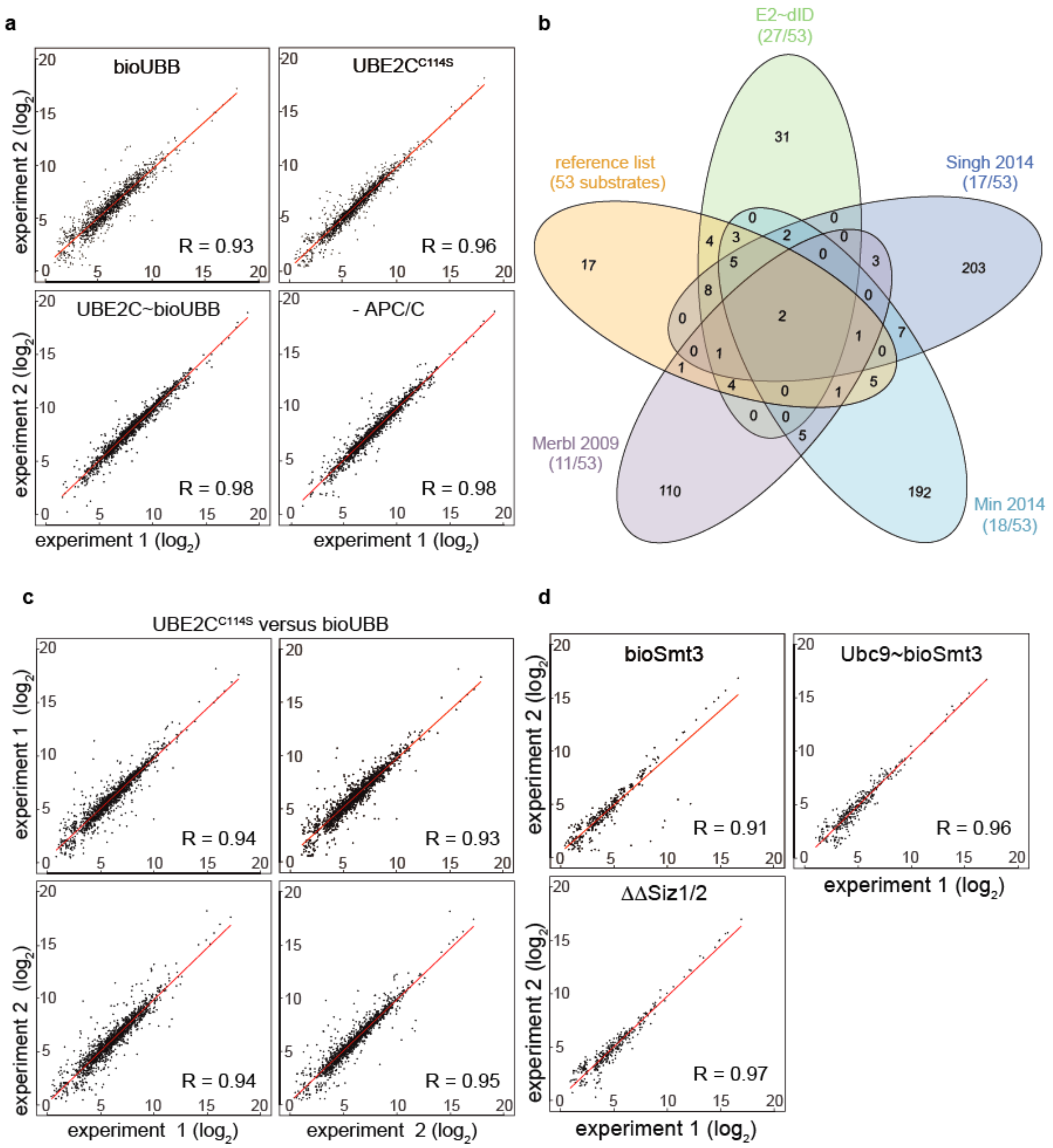

Supplementary Figure 3 | Reproducibility between independent E2~dID experiments. (**a**) Scatter plots showing $\log_2$-transformed TMT abundances and Pearson correlation coefficient for each E2~dID reaction with UBB and APC/C in between two independent experiments. (**b**) Venn diagram comparing the performance of E2~dID and three alternative approaches in identifying a reference list of 53 curated APC/C substrates with experimentally verified degrons (see also Supplementary Table 2). (**c**) Scatter plots showing $\log_2$-transformed TMT abundances and Pearson correlation coefficient of all UBE2C^{C114S} and bioUBB E2~dID control reactions in between two independent experiments. (**d**) Scatter plots showing $\log_2$-transformed TMT abundances and Pearson correlation coefficient for each E2~dID reaction with Smt3 and Siz1/Siz2 in between two independent experiments.

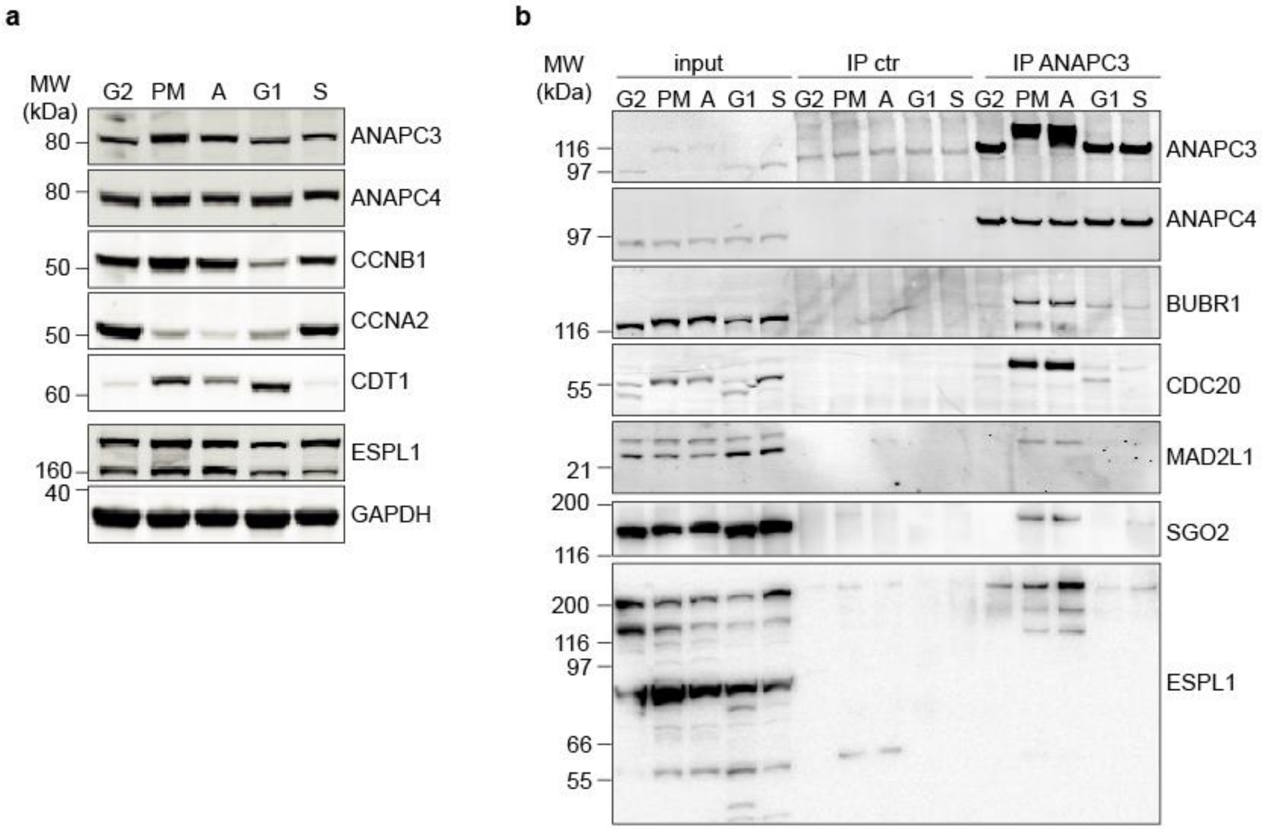

Supplementary Figure 4 | Interaction of SGO2 and ESPL1 with APC/C. (**a**) Representative Western blot analysis (n=3) of synchronized HeLa cell extracts with antibodies specific to cell cycle markers indicative for G2 phase; prometaphase (PM), anaphase (A), G1 phase and S phase. CCNB1 begins accumulating in late S phase and is degraded after PM, CCNA2 accumulates in S phase and is degraded in PM and A, CDT accumulates in G1 phase and is degraded upon entry into S phase. (**b**) Representative (n=3) analysis of control and ANAPC3 immunoprecipitations from synchronized cell extracts as in (a) showing the interaction of SGO2 and ESPL1 with APC/C predominantly in prometaphase (PM) and anaphase (A).

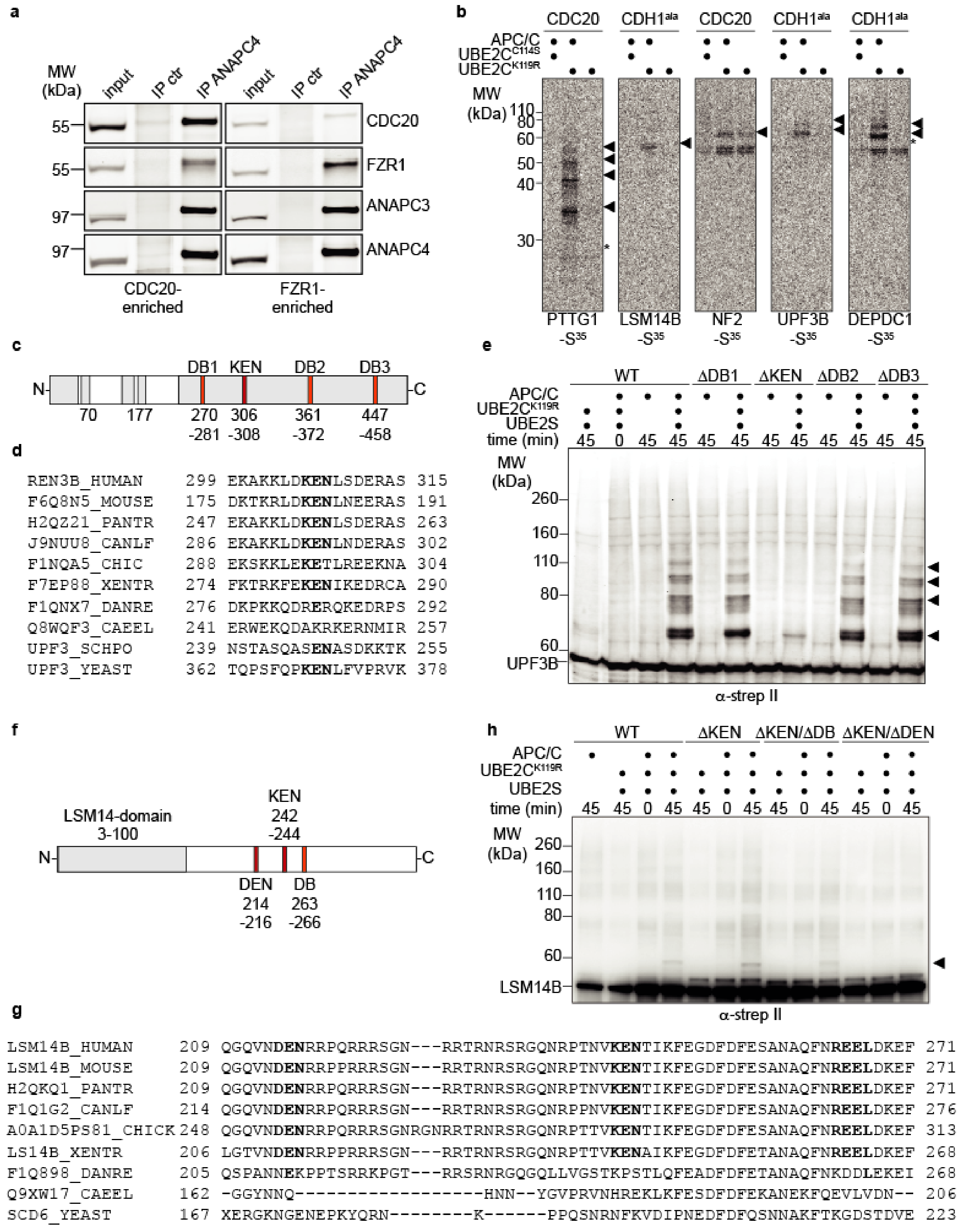

Supplementary Fig. 5: ***In vitro* reconstitution of UPF3B and LSM14B ubiquitination.**

(Figure legend on next page)

(**a**) Representative Western blot analysis (n=3) of control (ctr) and ANAPC4 immunoprecipitations showing the levels of CDC20 and FZR1 bound to APC/C in CDC20 and FZR1-enriched extracts (see methods). (**b**) Representative autoradiography (n=3) of NeutrAvidin-purified proteins from of E2~dID reactions using either CDC20 or FZR1-enriched extracts as in (a) in the presence of absence of APC/C. Arrow heads indicate bioUBB-modified substrates and asterisks co-purified unmodified substrates (**c**) Schematic presentation of human UPF3B indicating disordered regions (gray) and the relative positions of analyzed D boxes (DB) and the KEN box. (**d**) Sequence alignment of UPF3B (RENT3B) showing the conservation of the KEN box in different species. (**e**) Representative Western blot analysis (n=3) of an *in vitro* ubiquitination assay with recombinant strep II-tagged wild type (WT) and UPF3B degron mutants. Ubiquitin-modified species are marked with arrow heads. (**f**) Schematic representation of human LSM14B indicating the relative positions of analyzed KEN box, DEN box and the D box (DB). (**g**) Sequence alignment of LSM14B showing the conservation of the degron motifs in different species. (**h**) Representative Western blot analysis (n=4) of an *in vitro* ubiquitination assay with recombinant strep II-tagged wild type (WT) and LSM14B degron mutants. Note, LSM14B wildtype and mutants were supplied in the context of cleared *E. coli* lysates. Ubiquitin-modified species are marked with arrow heads.

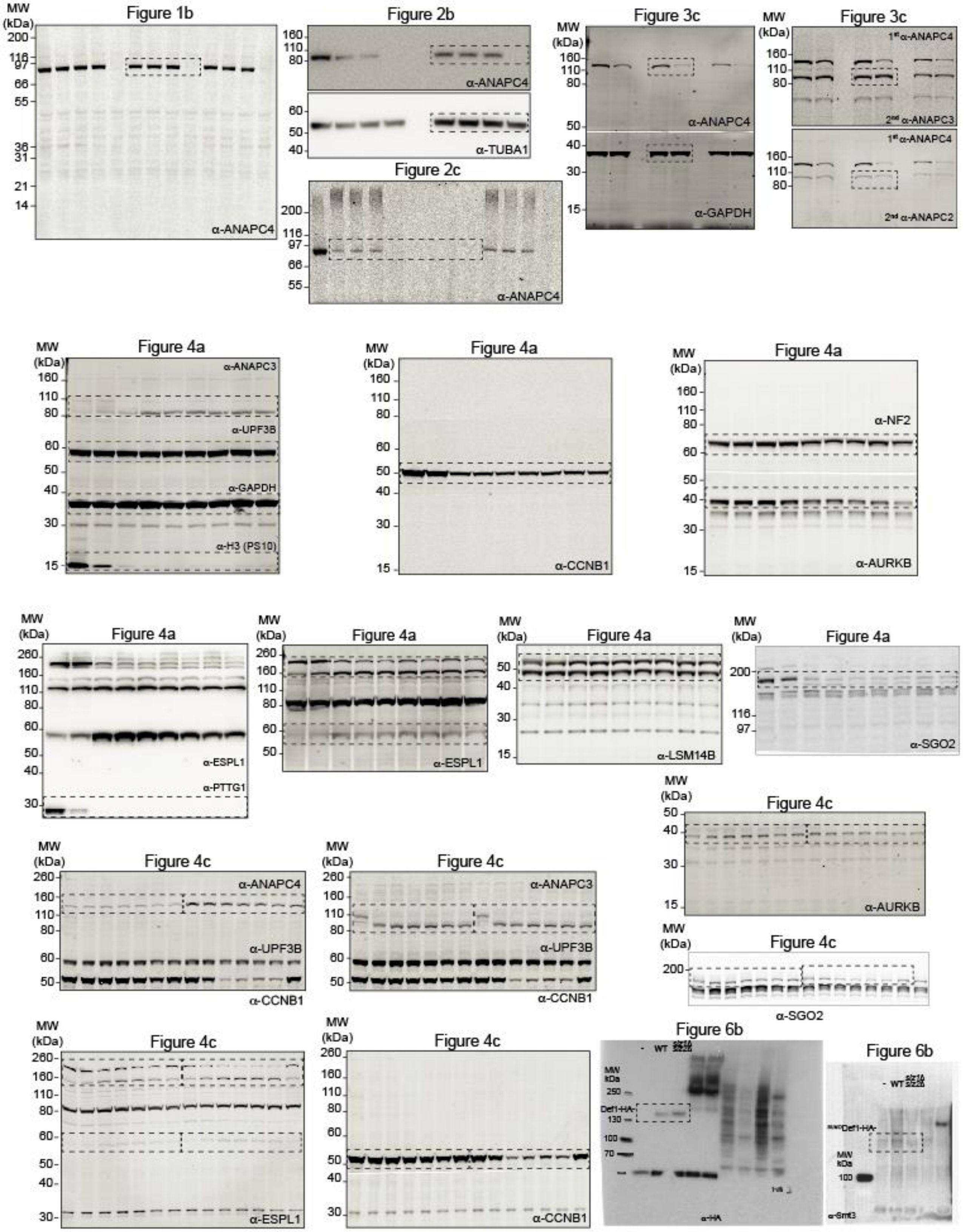

Supplementary Figure 6 | Complete scans of Western and SDS-PAGE analyses presented in Figures 1-4 and 6. Cropped regions are lined out by dashed boxes and the antibodies used for detection are indicated.
