# Supplementary material for: An E2-ubiquitin thioester-driven approach to identify substrates modified with ubiquitin and ubiquitin-like molecules
Source: Nat Commun. 2018 Nov 14;9:4776. doi: 10.1038/s41467-018-07251-5 (PMC6235928; doi:10.1038/s41467-018-07251-5)
Supplement: Supplementary file 3 — Description of Additional Supplementary Files [file 41467_2018_7251_MOESM3_ESM.pdf]

# Description of Additional Supplementary Files

**File Name:** Supplementary Data 1
**Description:** Ubiquitin E2~dID with the APC/C TMT mass spectrometry data of E2~dID experiments using bioUBB~UBE2C$^{K119R}$ thioesters with mock (+APC/C) and ANAPC4-depleted (-APC/C) HeLa K anaphase extracts. bioUBB and UBE2C$^{C114S}$ samples represent negative controls. Included as a separate tab are ubiquitination and APC/C degron analyses of identified candidates according to bioGRID and ProViz.

**File Name:** Supplementary Data 2
**Description:** Sensitivity and specificity of E2~dID with APC/C Sensitivity and specificity analyses of E2~dID with UBE2C and APC/C based on a list of curated mitotic substrates including references, APC/C degrons and APC/C co-activators responsible for recognition and ubiquitination. Included as separate tabs are data of alternative approaches (Co-regulation proteomics, mitotic exit proteome, protein microarrays) that were used to evaluate E2~dID and data used to generate the Venn diagram presented in Supplementary Figure 3b.

**File Name:** Supplementary Data 3
**Description:** diGly proteomics with and without APC/C TMT mass spectrometry data showing quantified di-glycine modified peptides with ratios from anaphase-like extracts with (+ APC/C) and without ANAPC4 (-APC/C).

**File Name:** Supplementary Data 4
**Description:** SUMO E2~dID with Siz1/Siz2 TMT mass spectrometry data of E2~dID experiments using bioSUMO~Ubc9 thioesters with extracts from wild type and siz1Δsiz2Δ S. cerevisae strains. Included as a separate tab is the SUMOylation state of identified candidates according to bioGRID.

**File Name:** Supplementary Data 5
**Description:** Sensitivity and specificity of E2~dID with Siz1/Siz2 Sensitivity and specificity analyses of E2~dID with SUMO and Siz1/Siz2 based on a list of curated Siz1/Siz2 substrates including references. Included as separate tabs are E2~dID candidates with information on their subcellular localization according to the Saccharomyces Genome Database.

**File Name:** Supplementary Data 6
**Description:** Plasmids and cloning List of plasmids employed in this study including information on used oligonucleotides to generate inserts, vector backbones, and the restriction sites used for cloning.

**File Name:** Supplementary Data 7
**Description:** Antibodies List of antibodies used in this study including information on the manufacturer, species, dilutions and the application within the study
